# Supplementary material for: Primary Care and Physical Literacy: A Non-Randomized Controlled Pilot Study to Combat the High Prevalence of Physically Inactive Adults in Austria
Source: Int J Environ Res Public Health. 2021 Aug 14;18(16):8593. doi: 10.3390/ijerph18168593 (PMC8394934; doi:10.3390/ijerph18168593)
Supplement: Supplementary file 1 [file ijerph-18-08593-s001.zip › Table S1 Detailed description of the domains and intervention approach.pdf]

Domains	Description	Exemplary Intervention Strategies
**Physical Activity Behavior** (behavioral domain)	• Refers to the extent to which an individual regularly engages in meaningful and personally challenging activities [1].<br>• If individuals exhibit a commitment for engagement in physical activities for life, they will show this by being physically active [2].	• Targeted indirectly by improvements in other physical literacy domains.<br>• Self-regulatory-skills: Goal setting, coping planning and self-monitoring worksheets [15].
**Motivation** (affective domain)	• Refers to a person's enthusiasm and pleasure in embracing physical activity as an integral part of life [1].<br>• Important predictor of initiation and maintenance of long-term physical activity adherence across all ages [3].	• Based on the self-determination theory [16].<br>• Established Strategies: Promote choice of activities and the development of social relationships as well as provide positive feedback [17].
**Knowledge** (cognitive domain)	• Refer to a person’s knowledge about how to perform a variety of physical activities ("how to move") as well as a person’s knowledge of the everyday relevance of exercises and the health benefits of physical activity in general [4].<br>• Associated with an individual’s physical activity level [5,6] and with other domains of physical literacy such as motivation [7,8].	• Can be imparted during exercises, recovery periods, or through the implementation of learning-oriented walking/running games [18].<br>• Established pedagogical and methodological principles: Proceed from simple to difficult, use a simple and pictorial language and provide continuous repetition [19-21].
**Attitude/Understanding** (affective domain)	• Refers to a person's positive and negative evaluation of being physically active and to a person’s understanding of the value of being physically active for life, respectively [4,9].<br>• A basis requirement to create a commitment to a physically active lifestyle [9,10].	• Targeted indirectly by improvements in other physical literacy domains, especially in the domain knowledge [22].<br>• Creating an intervention setting that enables empowering, enjoyable and motivational experiences of being physically active [23].
**Self-efficacy/Confidence** (affective domain)	• Refers to a person’s ability to perform and sustain feasible changes in one’s physical activity behavior as well as to a person’s ability to overcome physical activity barriers [11,12].<br>• Most robust predicator of long-term physical activity regardless of the sample population [13,14].	• Based on the self-efficacy theory [24].<br>• Established strategies: Mastery experience, vicarious experience and verbal persuasion [28].

References cited in the table

1. The International Physical Literacy Association. Consensus statement. Available online: https://physicalliteracy.ca/physical-literacy/consensus-statement/ (accessed 26 Jun 2021).
2. Gunnell, K. E.; Longmuir, P.; Barnes, J. D.; Belanger, K.; Tremblay, M. Refining the Canadian Assessment of Physical Literacy based on theory and factor analyses. *BMC Public Health* **2018**, 18. 10.1186/s12889- 018-5899-2.
3. Teixeira, P.J.; Carraça, E.V.; Markland, D.; Silva, M.N; Ryan, R.M. Exercise, physical activity, and self-determination theory: A systematic review. *Int J Behav Nutr Phys Act* **2012**, 9, https://doi.org/10.1186/1479-5868-9-78
4. Longmuir, P.E.; Woodruff, S.J.; Boyer, C.; Llody, M.; Tremblay, M.S. Physical Literacy Knowledge Questionnaire: feasibility, validity, and reliability for Canadian children aged 8 to 12 years. *BMC Public Health* **2018**, 18, https://doi.org/10.1186/s12889-018-5890-y
5. Rimal, R. N. Longitudinal Influences of Knowledge and Self-efficacy on Exercise Behavior: Tests of a Mutual Reinforcement Model. *J Health Psychol* **2015**, 6, 31–46. 10.1177/135910530100600103
6. Fitzgerald, J.T; Singleton, S.P; Neale, A.V; Prasad, A.S; Hess, J.W. Active level, fitness status, exercise knowledge, and exercise Beliefs among healthy, older African American and white women. *J Aging Health* **1994**, 6, 296–323.
7. Frikkel, J.; Götte, M.; Beckmann, M.; Kasper, S.; Hense, J.; Teufel, M.; Schuler, M.; Tewes, M. Fatigue, barriers to physical activity and predictors for motivation to exercise in advanced Cancer patients. *BMC Palliat Care* **2020**, 19. 10.1186/s12904-020-00542-z
8. Trost, S.G.; Saunders, R.; Ward, D.S. Determinants of Physical Activity in Middle School Children. *Am. J. Health Behav* **2002**, 26, 95-102. 10.5993/AJHB.26.2.2
9. Edwards, L.C.; Bryant, A.S.; Keegan, R.J.; Morgan, K.; Jones, A.M. Definitions, foundations and associations of physical literacy: a systematic review. *Sports Med* **2017**, 47. 113-126. 10.1007/s40279-016-0560-7
10. Whitehead, M. The history and development of physical literacy. *ICSSPE* **2013**, Bulletin 65, 22–8. Available online: https://www.icsspe.org/sites/default/files/bulletin65_0.pdf (accessed 14 Jun 2021).
11. Corbin, C.B. Implications of physical literacy for research and practice: A Commentary. *Res Q Exerc Sport* **2016**, 87, 14-27. 10.1080/02701367.2016.1124722
12. Jones, G.R.; Stathokostas, L.; Young, B.W.; Wister, A.V.; Chau, S.; Clark, P.; Duggan, M.; Mitchell, D.; Nordland, P. Development of a physical literacy model for older adults - a consensus process by the collaborative working group on physical literacy for older Canadians. *BMC Geriatr* **2018**, 18. 10.1186/s12877-017-0687-x
13. Rhodes, R.E; Janssen, I; Bredin, S.S.D; Warburton, D.E.R; Bauman, A. Physical activity: health impact, prevalence, correlates and interventions. *Psychol Health* **2017**, 32, 942–75. https://doi. org/10.1080/08870446.2017.1325486
14. Keller, C.; Fleury, J.; Gregor-Holt, N.; Thompson, T. Predictive Ability of Social Cognatic Theory in Exercise Research: An Integrated Literature Review. *Online J Knowl Synth Nurs* **1999**, 6.
15. Rhodes, R.E.; Pfaeffli, L.A.; Mediators of physical activity behaviour change among adult non-clinical populations: a review update. *Int J Behav Nutr Phys Act* **2010**, 7. https://doi.org/10.1186/1479-5868-7-37
16. Deci, E.L.; Ryan, R.M. *Intrinsic motivation and self-determination in human*. Plenum Publishing Co: New York, 1985.
17. Kilpatrick, M.; Herbert, E.P.; Jacobsen, D. Physical Activity Motivation: A Practitioner's Guide to Self-Determination Theory. *J Phys Edu Recreat Dance* **2002**, 73, 36-41. 10.1080/07303084.2002.10607789
18. Holler, P.; Jaunig, J.; Amort, F.M.; Tuttner, S.; Hofer-Fischanger, K.; Wallner, D.; Simi, H.; Müller, A.; van Poppel, M,N,M.; Moser, O. Holistic physical exercise training improves physical literacy among physically inactive adults: a pilot intervention study. *BMC Public Health* **2019**, 19, 393. 10.1186/s12889-019-6719-z
19. Weiss, B. D. Health Literacy and patient safety: Help patients understand. Manual for clinicians. Available online: https://www.ufjf.br/getmedicina/files/2015/11/BARRY-WEISS.pdf (accessed 26 Jun 2021).
20. Ebbinghaus, H. *Über das Gedächtnis - Untersuchungen zur experimentellen Psychologie*, Leipzig: Duncker & Humblot, **1885**.

21. Bräutigam, M. Sportdidaktik. Ein Lehrbuch in 12 Lektionen. 3rd ed.;Aachen: Meyer & Meyer, **2003**.
22. Whitehead, M.; Almond, L. Creating learning experiences to foster physical literacy. *ICSSPE* **2013**, Bulletin 65, 73-80. Available online: https://www.icsspe.org/sites/default/files/bulletin65_0.pdf (accessed 20 Jun 2021).
23. Almond, L. What is the relevance of physical literacy for adults? *ICSSPE* **2013**, Bulletin 65, 215-36. Available online: https://www.icsspe.org/sites/default/files/bulletin65_0.pdf (accessed 14 Jan 2021).
24. Bandura, A. Self-efficacy: Toward a unifying theory of behavioral change. *Psychol. Rev* **1977**, 84, 191-215. 10.1037/0033-295X.84.2.191
25. McAuley, E. Enhancing psychological health through physical activity. In *Towards Active Living: Proceedings of the International Conference on Physical Activity, Fitness and Health;* Quinney, H.A., Gauvin, L., Wall, A.E.T., Eds.; Human Kinetics: Champaign: Human Kinetics, 1994, pp. 83-90.
