# Supplementary material for: Primary Care and Physical Literacy: A Non-Randomized Controlled Pilot Study to Combat the High Prevalence of Physically Inactive Adults in Austria
Source: Int J Environ Res Public Health. 2021 Aug 14;18(16):8593. doi: 10.3390/ijerph18168593 (PMC8394934; doi:10.3390/ijerph18168593)
Supplement: Supplementary file 1 [file ijerph-18-08593-s001.zip › Table S2 Template for Intervention Description and Replication (TIDieR) checklist.pdf]

TIDieR
Template for Intervention Description and Replication

The TIDieR (Template for Intervention Description and Replication) Checklist*:

Information to include when describing an intervention and the location of the information

Item number	Item	Where located ** Primary paper (page or appendix number)	Other † (details)
	**BRIEF NAME**		
**1.**	Provide the name or a phrase that describes the intervention:	Page 3 (paragraph 2)	• Physical literacy-based intervention.
	**WHY**		
**2.**	Describe any rationale, theory, or goal of the elements essential to the intervention:	Page 2-3<br>Page 5-6 (section 2.5.)<br>Supplementary material (No. 2).	• Physical literacy is a multidimensional construct and hypothetical associated with life-long physical activity engagement.<br>• The intervention was designed to target all physical literacy domains simultaneously.<br>• A series of principles and established strategies (e.g. based on social cognitive theory or self-determination theory) were applied to elite improvements in all domains.

	**WHAT**		
**3.**	Materials: Describe any physical or informational materials used in the intervention, including those provided to participants or used in intervention delivery or in training of intervention providers. Provide information on where the materials can be accessed (e.g. online appendix, URL).	Page 5-6 (section 2.5.)	• Various training tools including resistance bands, gymnastic balls, and gymnastic mats.<br>• Print materials outlining the national physical activity guidelines or the benefits of being physically active.<br>• Worksheets focusing on physical activity goal setting, coping planning and self-monitoring.<br>• Print materials and worksheets are available on request from the corresponding author of the primary paper (peter.holler@fh-joanneum.at).<br>• Heart rate-monitors (Polar 610 and 810, Polar Electro OY, Kempele, Finland) and RPE-scales (to control/monitor exercise the intensity).
**4.**	Procedures: Describe each of the procedures, activities, and/or processes used in the intervention, including any enabling or support activities.	Page 5-6 (section 2.5.)	• Begin of each session: warm-up walking game (5min), mobilization period of major joints (5min).<br>• Main part (35min): session-specific exercises (i.e. strength or multimodal

	exercises) mixed with elements aimed at physical activity knowledge transfer.<br>• Strength-based sessions: simple body weight exercises and exercises with various training tools; during the recovery periods and also during the execution of the exercises, information about how to perform the exercises correctly and also about "everyday relevance" of each exercise as well as about the health benefits of aerobic and strength exercises and regular physical activity in general were provided.<br>• Strong focus on partner exercises to promote social interactions,<br>• Multimodal-based session (2 parts):<br>(i) learning-oriented walking/running (example available at additional file 2 in https://bmcpublichealth.biomedcentral.com/articles/10.1186/s12889-019-6719-z),<br>(ii) strength-related exercises taken from the strength-based sessions.<br>• End of each session (5min): providing positive feedback to participants, distributing and elaborating print materials and worksheets.

			• All messages to the participants were framed positively with a strong focus on the affective properties of physical activity
	**WHO PROVIDED**		
**5.**	For each category of intervention provider (e.g. psychologist, nursing assistant), describe their expertise, background and any specific training given.	Page 5-6 (section 2.5.)	• Sport scientist (male): five years of work experience, especially with inactive adults; additional a physical education teacher; no specific training given.
	**HOW**		
**6.**	Describe the modes of delivery (e.g. face-to-face or by some other mechanism, such as internet or telephone) of the intervention and whether it was provided individually or in a group.	Page 5-6 (section 2.5.)	• Each intervention days consisting of two consecutive 50-minute-group sessions, focusing either on strength-, or on multimodal (combination of strength- and aerobic) related activities.<br>• Participants were allowed to attend one or both sessions on one intervention day, according to their preferences.
	**WHERE**		
**7.**	Describe the type(s) of location(s) where the intervention occurred, including any necessary infrastructure or relevant features.	Page 3 (paragraph 2)<br>Page 4 (paragraph 2)<br>Page 5-6 (section 2.5.)	• Recruitment and interventions took place in Mürzzuschlag, a district of Styria, Austria.

			• All session took place in a gym of an elementary school (with standard equipment).
	**WHEN and HOW MUCH**		
**8.**	Describe the number of times the intervention was delivered and over what period of time including the number of sessions, their schedule, and their duration, intensity or dose.	Page 4 (paragraph 2)<br>Page 5-6 (section 2.5.)	• Target exercise intensity: moderate, heart rate: 64-76% of HR_{max}, RPE scale: 12-13.<br>• Start of the intervention: March 14, (spring).<br>• 14 intervention days in total, each consisting of two consecutive 50-minute-group sessions, focusing either on strength-, or on multimodal (5min warming up, 5min mobilization, 35min main part, 5min end of session).<br>• One intervention day per week (Wednesday); begin of the strength-based session, 05:30 pm; begin of the multimodal-based session. 07:00 pm.<br>• Participants were allowed to attend one or both sessions on one intervention day, according to their preferences.

	**TAILORING**		
**9.**	If the intervention was planned to be personalized, titrated or adapted, then describe what, why, when, and how.	Page 5-6 (section 2.5.)	• All exercises and games in both sessions were tailored to participants' physical and mental constitutions to ensure *mastery experience*, the most powerful influence on physical activity self-efficacy.<br>• Intensity was personalized based on each participant predicted HF_{max} [HR_{max} = 208 – (0.7 * age)]<br>• At the end of each session participants were given individual, positive and encouraging feedback, as a further strategy to enhance physical activity self-efficacy.
	**MODIFICATIONS**		
**10.‡**	If the intervention was modified during the course of the study, describe the changes (what, why, when, and how).		• N/A
	**HOW WELL**		
**11.**	Planned: If intervention adherence or fidelity was assessed, describe how and by whom, and if any strategies were used to maintain or improve fidelity, describe them.	Page 5-6 (section 2.5.)	• Adherence to the intervention was calculated by the ratio between attended intervention days divided by theoretical

			possible intervention days (given as percentage).<br>• Adherence were assessed by the Sport scientist, who led the intervention.<br>• The intervention was designed per se to ensure a high adherence (e.g. by allowing participants to attend one or both sessions on one intervention day).
**12.‡**	Actual: If intervention adherence or fidelity was assessed, describe the extent to which the intervention was delivered as planned.	Page 9 (section 3.2)	• Total average adherence to the intervention was 68 ± 20%.

** **Authors** - use N/A if an item is not applicable for the intervention being described. **Reviewers** – use '?' if information about the element is not reported/not sufficiently reported.

† If the information is not provided in the primary paper, give details of where this information is available. This may include locations such as a published protocol or other published papers (provide citation details) or a website (provide the URL).

‡ If completing the TIDieR checklist for a protocol, these items are not relevant to the protocol and cannot be described until the study is complete.

* We strongly recommend using this checklist in conjunction with the TIDieR guide (see *BMJ* 2014;348:g1687) which contains an explanation and elaboration for each item.

* The focus of TIDieR is on reporting details of the intervention elements (and where relevant, comparison elements) of a study. Other elements and methodological features of studies are covered by other reporting statements and checklists and have not been duplicated as part of the TIDieR checklist. When a **randomised trial** is being reported, the TIDieR checklist should be used in conjunction with the CONSORT statement (see www.consort-statement.org) as an extension of **Item 5 of the CONSORT 2010 Statement.** When a **clinical trial protocol** is being reported, the TIDieR checklist should be used in conjunction with the SPIRIT statement as an extension of **Item 11 of the SPIRIT 2013 Statement** (see www.spirit-statement.org). For alternate study designs, TIDieR can be used in conjunction with the appropriate checklist for that study design (see www.equator-network.org).
